# Extension of the short wavelength side of fluorescent proteins using hydrated chromophores, and its application

**DOI:** 10.1038/s42003-022-04153-7

**Published:** 2022-11-03

**Authors:** Kazunori Sugiura, Takeharu Nagai

**Affiliations:** SANKEN (The Institute of Scientific and Industrial Research), Osaka University, Ibaraki, Osaka Japan

**Keywords:** Biophysics, Molecular engineering

## Abstract

To perform correlation analysis between different physiological parameters using fluorescent protein-based functional probes, diversification of wavelength properties of fluorescent proteins is underway. However, the shortest emission wavelength of fluorescent proteins has not been updated for more than 10 years. Here, we report the development of Sumire, a fluorescent protein emitting 414 nm violet fluorescence from a hydrated chromophore. The Sumire’s fluorescence property allows for the creation of FRET probes that can be used simultaneously with CFP-YFP based FRET probes for multi-parameter analysis.

## Abstract (web summary)

A violet fluorescent protein with emission wavelength 414 nm is engineered.

## Introduction

Most of the short-wavelength mutants categorized as cyan fluorescent protein and blue fluorescent protein were developed by replacing the 66th tyrosine of *Aequorea victoria* green fluorescent protein (avGFP), which constructs a GFP chromophore, with other aromatic amino acids such as tryptophan and histidine^1^. In the case of Sirius, the shortest emission variant within the currently available fluorescent proteins, the tyrosine, is replaced with phenylalanine^2^. Similar to avGFP, a short-wavelength mutant of mNeonGreen, called NeonCyan has been created by replacing tyrosinein the chromophore with tryptophan^3^. The replacement of the 66th amino acid is a reasonable approach, but the limited availability of natural aromatic amino acids requires a different approach for the creation of other short-wavelength fluorescent proteins.

The properties of fluorescent proteins are not determined by the chromophore alone but are strongly influenced by the interaction of the chromophore with the surrounding amino acids and water molecules. For example, mKalama1, an avGFP mutant, emits blue fluorescence peaking at 456 nm, although it has a chromophore similar to that of avGFP (Ser-Tyr-Gly)^4^. When avGFP is excited by 400 nm light, a proton is transferred from the neutral chromophore to the E222 residue through a water molecule and the S205 (or T203) residue, and the chromophore is ionized before emission. This phenomenon is referred as excited state proton transfer (ESPT)^5,6^. In mKalama1, the chromophore is kept in a neutral form and shows blue emission because the ESPT pathway was removed by two mutations, T203V and S205V. Additionally, the V224R mutation derived from azurite^7^, a BFP variant, was added to stabilize the chromophore^7^. Photoswitching, in which fluorescent proteins are switched on and off by irradiation with light at specific wavelengths, is also an interesting phenomenon caused by the interaction between chromophores and surrounding amino acids. Dreiklang, a YFP-based reversibly photoswitchable fluorescent protein, has two absorption peaks around 405 nm and 515 nm in the on-state and changes to the off-state when excited at 405 nm. In the off-state, Dreiklang has an absorption peak at approximately 340 nm^8^. Crystal structure analysis revealed that the 340 nm absorption originates from the hydrated YFP chromophore, and it has been reported that the E222 residue plays an important role in the hydration and dehydration of the chromophore^8–10^.

Therefore, we attempted to create mutants with shorter fluorescence wavelengths by controlling the interaction between the chromophore and the surrounding amino acid residues, rather than by replacing aromatic amino acids in the chromophore. As a result, we succeed in development of “Sumire”, a violet fluorescent protein that fluoresces at 414 nm, the shortest wavelength of any fluorescent protein reported to date.

## Result

### The protein design of Sumire

**Fig. 1:**
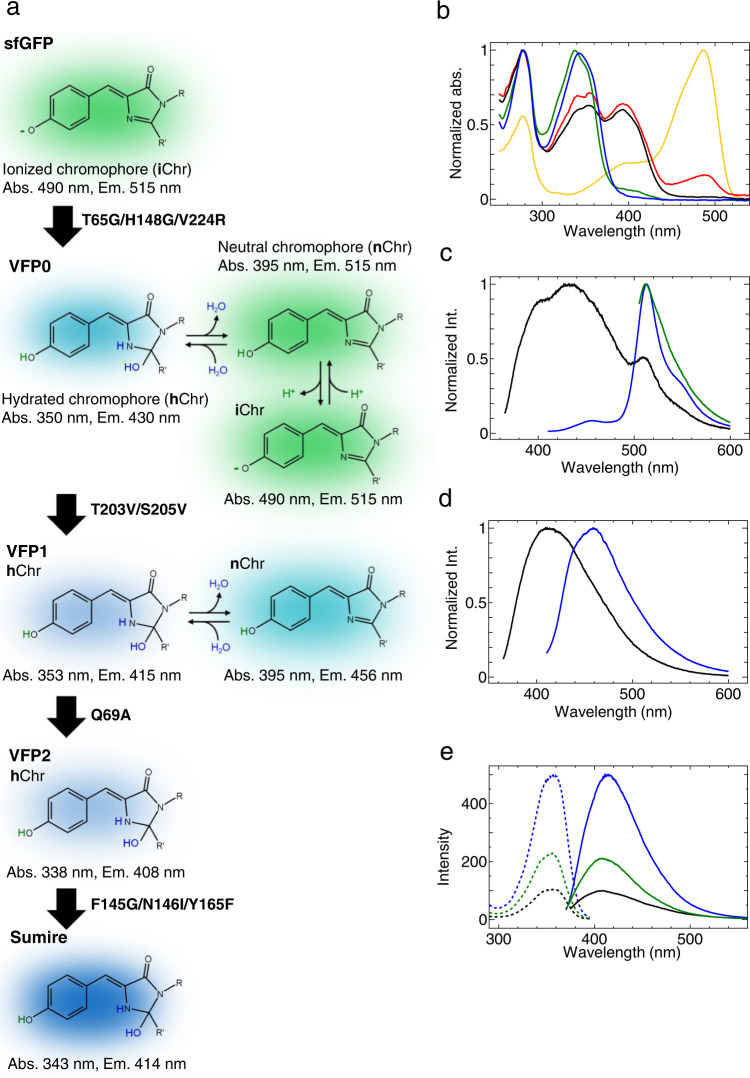
Designs and spectral properties of VFPs. **a** Schematic diagram of expected chromophore states of Sumire and parent proteins. **b** Normalized absorption spectrum of sfGFP (yellow), VFP0 (red), VFP1 (black), VFP2 (green), and Sumire (blue). **c** Normalized emission spectra of VFP0 excited by 350 nm (black), 395 nm (blue), and 490 nm (green). **d** Normalized emission spectra of VFP1 excited by 350 nm (black) and 395 nm (blue). **e** Excitation (dash line) and emission (solid line) spectra of VFP1 (black), VFP2 (green), and Sumire (blue). Spectra data are average measurements of independently prepared samples (*n* = 3).

Super folder GFP (sfGFP), an avGFP mutant with improved structural stability^11^, was used as the starting material. The T65G mutation was introduced to convert the chromophore to the YFP type. Then, H148G and V224R mutations were introduced by referring to mKalama1 to improve the emission intensity from the neutral chromophore (Fig. 1a). This mutant, notated as VFP0 (sfGFP T65G/H148G/V224R), has three absorption peaks around 350 nm, 395 nm, and 490 nm, in addition to absorption at 280 nm from aromatic amino acids (Fig. 1b, red line). These three absorption wavelengths were expected to originate from the hydrated chromophore (hChr), neutral chromophore (nChr), and ionized chromophore (iChr), respectively (Fig. 1a). VFP0 showed 515 nm green emission when excited by 395 nm and 490 nm, and 430 nm blue emission by 350 nm excitation (Fig. 1c). T203 and S205 were then replaced by valine to remove the ESPT pathway. This mutant, notated as VFP1, lost absorption near 490 nm (Fig. 1b, black line). Since the exclusion of the ESPT pathway resulted in a blue shift of the emission peaks, VFP1 emitted violet fluorescence of 415 nm and blue fluorescence with a 456 nm peak by 350 nm and 395 nm excitation, respectively (Fig. 1d). Next, we attempted to stabilize the hydrated chromophores. Since the Q69 forms a hydrogen bond with the E222 residue, which catalyzes the addition and removal of water molecules to and from the chromophore^9,10^ via a water molecule, mutations at this site were expected to affect the balance between hydration and dehydration of the chromophore. Therefore, we attempted to induce several mutations at this position and found that the Q69A mutation stabilized the hydrated chromophore. The VFP1 Q69A mutant, VFP2, had a single absorption peak at 340 nm (Fig. 1b, green line). Finally, three more mutations, Y145G/N146I and F165Y were introduced to improve fluorescence quantum yield by referring to Sirius. This new mutant also had a single absorption peak at 340 nm and showed 414 nm emission that was twice as bright as VFP2 (Fig. 1b blue line, Fig. 1e). We named this mutant “Sumire” after the Japanese word for “violet”. The mutation points for Sumire are shown in Supplementary Fig. 1. The absorption coefficient and quantum yield of Sumire were 2.0 × 10^4 ^M^−1^cm^−1^ and 0.70, respectively (Table 1). The fluorescence brightness of Sumire calculated from these values was approximately 3.9 times higher than that of Sirius (Table 1). Sumire had a low p*K*_a_ of 3.8 and emitted stable fluorescence over a wide pH range from 5.5 to 9.0. (Supplementary Fig. 2). In contrast, VFP0 and VFP1, which can take multiple chromophore states, showed strong pH dependence probably because the chromophore states are affected by pH (Supplementary Fig. 2). In HeLa cells, Sumire showed 3.3 times brighter emission than Sirius (Supplementary Fig. 3) and localized normally within specific cellular compartment by fusing it to various localization signal peptides and proteins (Supplementary Fig. 4).

**Table 1:** Fluorescence propaties.

Name	Mutations	Chromophore	λ_ab_^a^(nm)	λ_em_^b^(nm)	ε ^c^(×10^3 ^M^−1^cm^−1^)	φ^d^	Brightness^e^
VFP0	sfGFP+T65G/H148G/V224R	hChr	350	430	9.9	0.13	1.3
		nChr	395	515	9.2	0.11	1
		iChr	490	515	2.2	0.19	0.4
VFP1	VFP0 + T203V/S205V	hChr	354	410	13	0.13	1.7
		nChr	395	456	12	0.06	0.7
VFP2	VFP1 + Q69A	hChr	338	408	20	0.38	7.6
Sumire	VFP2 + Y145G/N146I/F165Y	hChr	343	414	20	0.70	14
Sirius	-	-	355	424	15	0.24	3.6

^a^Absorption peak.

^b^Emission peak.

^c^Absorption coefficient.

^d^Fluorescence quantum yield.

^e^(ε×ϕ).

### The development of functional indicator using Sumire

**Fig. 2:**
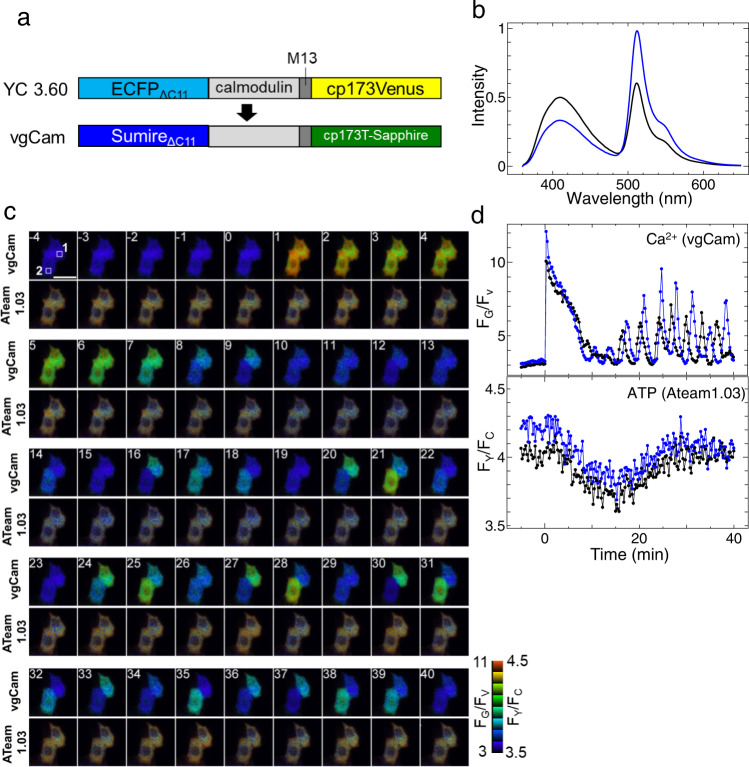
Sumire-T-sapphire based FRET probe. **a** Diagram of vgCam construct. **b** Emission spectra of vgCam in the presence of 20 μM CaCl_2_ (blue) and 10 mM EGTA (black). Spectra data are average measurements of independently prepared samples (*n* = 3). **c** A series of pseudo color ratio images of vgCam and ATeam1.03. Scale bar: 40 μm. **d** Time course of fluorescence intensity ratio of vgCam and ATeam1.03 inside ROI1 (black) and ROI2 (blue) depicted in the top-left image of **c**. 100 μM histamine was added at time 0.

Next, we attempted to develop a FRET-type functional indicator using Sumire. Sumire has a fluorescence peak at even shorter wavelengths than Sirius and therefore can be used as a FRET donor to pair with T-Sapphire^12^ (Supplementary Fig. 5a). The spectral overlap between Sumire and T-Sapphire (*J* = 3.1 × 10^−14 ^M^−1^ cm^3^) is 1.8 times larger than when Sirius is used as a donor (*J* = 1.7 × 10^−14 ^M^−1^ cm^3^). In addition, assuming κ^2^ = 2/3, the Förster distance (*R*_*0*_) of the Sumire-T-sapphire pair is (*R*_*0*_ = 4.0 nm) longer than that of the Sirius-T-sapphire pair (*R*_*0*_ = 3.0 nm). The ECFP and cp173Venus in the Ca^2+^ indicator, yellow cameleon 3.60 (YC 3.60)^13^, were substituted with Sumire and T-sapphire, respectively (Fig. 2a). In YC 3.60, cp173 Venus, a circular permuted mutant of YFP, was used as a FRET acceptor to optimize the relative angle between the chromophores^13^. Eleven amino acids from the C-terminal side of ECFP, the donor of YC 3.60, was removed because this region of avGFP mutants does not adopt a fixed structure and reduces the signal change rate of the indicator^13^. With reference to these points, we developed a Sumire-T-Sapphire pair-based Ca^2+^ indicator, “vgCam”. Recombinant vgCam showed a 2.4-fold change in the fluorescence intensity ratio of 510 nm to 414 nm with and without Ca^2+^ (Fig. 2b). The Sumire-T-Sapphire pair and the CFP-YFP pair have a small overlap in absorption spectra, so they can be excited independently using 350 nm and 440 nm as the excitation light (Supplementary Fig. 4b). As a demonstration, vgCam and the ATP indicator ATeam 1.03^14^ were co-expressed in HeLa cells, in the presence of histamine. vgCam and Ateam 1.03 showed signal changes independently (Fig. 2c, d). Thus, it is possible to create color variants of existing CFP-YFP-type FRET indicators and use them for multi-parameter simultaneous observation analysis of living cells using Sumire.

## Discussion

In this study, we developed a violet fluorescent protein “Sumire”, which updates the shortest fluorescence wavelength by utilizing the shortening of the π-conjugated system due to the hydration of the chromophore. In addition to chromophore hydration, the exclusion of the ESPT pathway, which is inherent to avGFP, is another major reason for the shorter emission wavelength of Sumire. Recently, a fluorescent protein whose chromophore contains only oxidized tyrosine without an imidazole ring was reported^15^. The fluorescent protein “bfVFP” is excited by light at 323 nm and emits fluorescence at 430 nm, a slightly longer wavelength than Sirius^15^. Because part of the imidazole ring is included in the π-conjugated system, Sumire has a longer absorption wavelength than bfVFP. However, the exclusion of the ESPT pathway in Sumire suppresses the red shift in fluorescence, which is probably the reason that the emission wavelength of Sumire is shorter than that of bfVFP.

We demonstrated a method for creating FRET-type indicators for multi-parameter simultaneous observation by using Sumire and T-Sapphire. Since Sumire and T-Sapphire are mutants created from avGFP as with CFP and YFP, it is expected that these mutants have very similar structures to each other. Therefore, by using the design of existing probes based on the CFP-YFP pair for the Sumire-T-Sapphire pair, it is possible to create emission color variations of existing probes in a relatively short term and perform simultaneous multi-parameter analysis.

Since hydration of the chromophore potentially occurs in chromophores by replacing the 66th tyrosine with another aromatic amino acids, the strategy used in this study may be useful for further multi-colorization of fluorescent proteins in the future. It may also be possible to further shorten the wavelength by applying the removal of the ESPT pathway to the aforementioned bfVFP.

## Methods

### Gene Construct

The pRSET_B_ vector encoding sfGFP was used as the starting material for the development of Sumire cells. All point mutations were introduced by fusing PCR products amplified with primers containing the mutations using the hot-fusion method^16^. For Sirius, ECFP and Venus cloned into the pRSET_B_ vector was used for E. coli expression. For experiments in HeLa cells, The cDNA of Sumire were subcloned into the pcDNA3 vector using hot fusion. For the P2A assay, cDNA of mCherry and P2A sequence, Sumire/ Sirius were fused in this order and subcloned into the pcDNA3 using hot fusion method. For the vgCam construct, pREST_B_ encoding YC 3.60, was used as a template for the sensor domain. Fluorescent protein sequences and sensor domain sequences amplified by PCR were fused to the pRSET_B_ and pcDNA3 vector sequences by hot fusion. The vgCam construct for E. coli expression had a Streptag added to the C-terminal side. The plasmids used to confirm localization were amplified by PCR using Sumire and each signal peptide or protein coding sequence, and connected to the pcDNA3 vector sequence using the hot fusion method.

### Protein expression and purification

To yield the recombinant protein, *E. coli* [JM109 (DE3)] was transformed using pRSET_B_ vectors encoding each product and cultured in 200 mL liquid LB medium at 23°C for 60-72 hours. *E. coli* was collected by centrifugation, resuspended in buffer containing 50 mM Tris-HCl (pH 8.0) and 20 mM imidazole, and crushed using a French press. Samples of the supernatant fraction and pellet were used to confirm that Sumire solubilizes to the same extent as sfGFP by SDS pages (Supplementary Fig. 6a). The supernatant of the centrifuged bacterial crushing solution was adsorbed onto Ni-NTA resin in an open column, washed with buffer for resuspension, and eluted with buffer containing 250 mM imidazole. Finally, the buffer was replaced with 50 mM MOPS (pH-7.2) and 50 mM KCl, using PD10. Regarding recombinant vgCam, after Ni-NTA purification, the eluted fraction was mixed with Strep-Tactin Superflow Plus (QIAGEN), washed with 50 mM Tris-HCl buffer on an open column, and eluted with 50 mM Tris-HCl buffer containing 2.5 mM desthiobiotin. Thereafter, the protein was buffer exchanged with PD10, similar to the other recombinant proteins. The purified samples were confirmed by SDS page to contain the target protein and the fluorescent protein whose main chains were cleaved before the chromophore as reported for RFP and others^17^ (Supplementary Fig. 6b). The thin band just below the main band of FP is likely derived from FP fragments, which is often observed in avGFP mutants.

### In vitro spectral analysis

The fluorescence spectra, absorption spectra, and fluorescence quantum yields were measured using F7000 (HITACHI), U-3900(HITACHI), and Quantaurus-QY (HAMAMATSU), respectively, in a buffer containing 50 mM MOPS (pH 7.2) and 50 mM KCl at room temperature. The pH titration was measured using a multimode plate reader, Spectra Max iD5 (Molecular Devise).

### Calculations of parameters

Spectra overlap (*J*) and Förster distance (*R*_*0*_) were calculated from the Förster equation.

1 $$J=\int {f}_{D}({{{{{\rm{\lambda }}}}}}){\varepsilon }_{A}({{{{{\rm{\lambda }}}}}}){{{{{{\rm{\lambda }}}}}}}^{4}d{{{{{\rm{\lambda }}}}}}$$

2 $${{R}_{0}}^{6}=9{Q}_{0}(ln10){\kappa }^{2}J/(128{\pi }^{5}{n}^{4}{N}_{A})$$

here, *f*_*D*_ (λ) and *ε*_*A*_ (*λ*) are the normalized emission spectra of the donor and the absorption spectra of the acceptor at wavelength λ, respectively. *Q*_0_ is the donor fluorescence quantum yield in the absence of an acceptor. κ, n, and *N*_A_ indicate the bipolar moment, refractive index of the solvent, and Avogadro’s number, respectively.

The ligand binding rates of FRET-based indicators (R_B_) were calculated using Eq. 3.

3 $${{{{{{\rm{R}}}}}}}_{{{{{{\rm{B}}}}}}}=({{{{{\rm{R}}}}}}-{R}_{n})/({R}_{s}-{R}_{n})$$

here, R, *R*_*n*_ and *R*_*s*_ are the signal intensity ratios of the acceptor to donor at the indicated concentrations in the absence and presence of a saturating concentration, respectively.

The dissociation constant (*K*_*D*_) and Hill coefficient (*n*) were derived by fitting R_B_ with Hill’s equation (Eq. 4).

4 $${{{{{{\rm{R}}}}}}}_{{{{{{\rm{B}}}}}}}={[{{{{{\rm{L}}}}}}]}^{n}/({{K}_{D}}^{n}+{[L]}^{n})$$

here, [L] is ligand concentrations.

### Cell culture and transfection

HeLa cells were grown in Dulbeccos’s modified Eagle’s medium (Wako) containing 10% fetal calf serum. One day before transfection, the cells were dissociated and transferred to a 35 mm glass-bottom dish. Transfection with pcDNA cording each fluorescent protein was done by using polyethylene imine (PEI Max 40 K; Polyscience) according to the manufacturer’s instruction.

### Imaging

For cell imaging, an epifluorescence microscope Ti-II (Nikon) with a control program, Fusion (Andor), was used. CFI Plan Fluor 60XS Oil (Nikon) was used as the objective lens because the transmission of 350 nm excitation light was better. Fluorescence images were captured using an EM CCD camera (iXon Ultra; Andor). For observations using Sumire, a dichroic mirror (FF365-Di01-25×36; Semrock), an excitation filter (F01-334/40-25; Semrock), and an emission filter (FF01-417/60-25; Semrock) were used. A 340 nm LED line of LEDhub (Omicron) was used as the excitation light. To observe the blue fluorescence from VFP1, a dichroic mirror (Di01-R405/488/561/635; Semrock), an excitation filter (FF01-405/10-25; Semrock), emission filters (FF02-447/60-25; Semrock), and a 400 nm LED line of LEDhub were used. In the P2A assay, the emission filters were replaced with filters (FF01-440/40-25; Semrock) optimized for Sirius rather than for Sumire. For mCherry observation, a dichroic mirror (FF593-Di03-25×36; Semrock), an excitation filter (FF01-562/40-32; Semrock), fluorescence filters (FF02-641/75-32; Semrock), and a 550 nm line of an LED light source, Niji (blue box optics), were used. For FRET imaging using a Sumire-T-sapphire pair, the excitation light source, dichroic mirror, and excitation filter were the same as those in the Sumire observation above. The emission filter was switched between the violet channel (FF01-417/60-25; Semrock) and green channel (FF02-520/28-32; Semrock). For FRET imaging using Ateam 1.03, a dichroic mirror (FF458-Di02-32×44; Semrock), an excitation filter (FF01-425/30-25; Semrock), and a 455 nm LED line of LEDhub were used. The emission filter was switched between the cyan channel (FF01-483/32-32) and the yellow channel (FF01-542/27-32; Semrock). Immediately prior to observation, the medium for cell culture was replaced with that for observation (F12/DMEM; Gibco). During imaging, samples were maintained in a 37 °C, 5% CO_2_ environment in a stage-top incubator, STX (TOKAI HIT).

### Statistics and reproducibility

For the in vitro experiments, three independent samples of the same lot of purified protein were prepared and each sample was measured. For dual FRET experiments under a microscope, it is not appropriate to give an average value because the timing of calcium oscillation varies for each individual cell. Therefore, we only showed the changes within a specific ROI for individual cells shown in Fig. 2c.

### Reporting summary

Further information on research design is available in the Nature Research Reporting Summary linked to this article.

## Supplementary information

Supplemental Information

Description of Additional Supplementary Files

Supplementary Data 1

Reporting Summary

## Data Availability

Data and materials supporting this study are available from the authors upon reasonable request. Plasmid constructs of Sumire (Addgene ID, 193361) and vgCam (Addgene ID, 193362) are available through Addgene. Original data for Figs. 1a–e, 2b, d, S2a–d, S3b, S5a, b are provided as Supplementary Data 1. The purity of the recombinant proteins used in the experiments was checked on the SDS page (Supplementary Fig. 6).
