# Supplementary material for: Extension of the short wavelength side of fluorescent proteins using hydrated chromophores, and its application
Source: Commun Biol. 2022 Nov 3;5:1172. doi: 10.1038/s42003-022-04153-7 (PMC9633818; doi:10.1038/s42003-022-04153-7)
Supplement: Supplementary file 1 — Supplemental Information [file 42003_2022_4153_MOESM1_ESM.pdf]

Extension of the short wavelength side of fluorescent proteins using hydrated chromophores, and its application

Supplemental Information

a

```
                 10         20         30         40         50         60         70         80
avGFP     MSKGEELFTG VVPILVELDG DVNGHKFSVS GEGEGDATYG KLTLKFICTT GKLPVPWPTL VTTFSYGVQC FSRYPDHMKQ
BFP       MSKGEELFTG VVPILVELDG DVNGHKFSVS GEGEGDATYG KLTLKFICTT GKLPVPWPTL VTTFSHGVQC FSRYPDHMKQ
CFP       MSKGEELFTG VVPILVELDG DVNGHKFSVS GEGEGDATYG KLTLKFICTT GKLPVPWPTL VTTFSWGVQC FSRYPDHMKQ
Sirius   MVSKGEELFTG VVPILVELDG DVNGHRFSVS GEGEGDATYG KLTLKLICTT GKLPVPWPTL VTTLQFGVLC FARYPDHMKQ
mKalama1 MVSKGEELFTG VVPILVEMDG DVNGRKFSVR GVGEGDATHG KLTLKFICTS GKLPVPWPTL VTTLSYGVQC FSRYPDHMKQ
sfGFP    MVSKGEELFTG VVPILVELDG DVNGHKFSVR GEGEGDATNG KLTLKFICTT GKLPVPWPTL VTTLTYGVQC FSRYPDHMKR
VFP0     MVSKGEELFTG VVPILVELDG DVNGHKFSVR GEGEGDATNG KLTLKFICTT GKLPVPWPTL VTTLGYGVQC FSRYPDHMKR
VFP1     MVSKGEELFTG VVPILVELDG DVNGHKFSVR GEGEGDATNG KLTLKFICTT GKLPVPWPTL VTTLGYGVQC FSRYPDHMKR
VFP2     MVSKGEELFTG VVPILVELDG DVNGHKFSVR GEGEGDATNG KLTLKFICTT GKLPVPWPTL VTTLGYGVAC FSRYPDHMKR
Sumire   MVSKGEELFTG VVPILVELDG DVNGHKFSVR GEGEGDATNG KLTLKFICTT GKLPVPWPTL VTTLGYGVAC FSRYPDHMKR

                 90        100        110        120        130        140        150        160
avGFP     HDFFKSAMPE GYVQERTIFF KDDGNYKTRA EVKFEGDTLV NRIELKGIDF KEDGNILGHK LEYNYNSHNV YIMADKQKNG
BFP       HDFFKSAMPE GYVQERTIFF KDDGNYKTRA EVKFEGDTLV NRIELKGIDF KEDGNILGHK LEYNFNSHNV YIMADKQKNG
CFP       HDFFKSAMPE GYVQERTIFF KDDGNYKTRA EVKFEGDTLV NRIELKGIDF KEDGNILGHK LEYNYNSHNV YIMADKQKNG
Sirius    HDFFKSAMPE GYVQERTIFF KDDGNYKTRA EVKFEGDTLV NRIELKGIDF KEDGNILGHK LEYNGISSNV YITADKQKNG
mKalama1  HDFFKSAMPE GYVQERTIFF KDDGSYKTRA EVKFEGDTLV NRIVLKGTDF KEDGNILGHK LEYNMNVGNV YITADKQKNG
sfGFP     HDFFKSAMPE GYVQERTISF KDDGTYKTRA EVKFEGDTLV NRIELKGIDF KEDGNILGHK LEYNFNSHNV YITADKQKNG
VFP0      HDFFKSAMPE GYVQERTISF KDDGTYKTRA EVKFEGDTLV NRIELKGIDF KEDGNILGHK LEYNFNSGNV YITADKQKNG
VFP1      HDFFKSAMPE GYVQERTISF KDDGTYKTRA EVKFEGDTLV NRIELKGIDF KEDGNILGHK LEYNFNSGNV YITADKQKNG
VFP2      HDFFKSAMPE GYVQERTISF KDDGTYKTRA EVKFEGDTLV NRIELKGIDF KEDGNILGHK LEYNFNSGNV YITADKQKNG
Sumire    HDFFKSAMPE GYVQERTISF KDDGTYKTRA EVKFEGDTLV NRIELKGIDF KEDGNILGHK LEYNGISGNV YITADKQKNG

                170        180        190        200        210        220        230
avGFP     IKVNFKIRHN IEDGSVQLAD HYQQNTPIGD GPVLLPDNHY LSTQSALSKD PNEKRDHMVL LEFVTAAGIT HGMDELYK
BFP       IKVNFKIRHN IEDGSVQLAD HYQQNTPIGD GPVLLPDNHY LSTQSALSKD PNEKRDHMVL LEFVTAAGIT HGMDELYK
CFP       IKVNFKIRHN IEDGSVQLAD HYQQNTPIGD GPVLLPDNHY LSTQSALSKD PNEKRDHMVL LEFVTAAGIT HGMDELYK
Sirius    IKAHFKIRHN IEDGGVQLAD HYQQNTPIGD GPVLLPDNHY LSVQSKLSKD PNEKRDHMVL LESVTAAGIT LGMDELYK
mKalama1  IKANFEIRHN VEDGGVQLAD HYQQNTPIGD GSVLLPDNHY LSVQVKLSKD PNEKRDHMVL LEFRTAAGIT PGMDELYK
sfGFP     IKANFKIRHN VEDGSVQLAD HYQQNTPIGD GPVLLPDNHY LSTQSVLSKD PNEKRDHMVL LEFVTAAGIT HGMDELYK
VFP0      IKANFKIRHN VEDGSVQLAD HYQQNTPIGD GPVLLPDNHY LSTQSVLSKD PNEKRDHMVL LEFRTAAGIT HGMDELYK
VFP1      IKANFKIRHN VEDGSVQLAD HYQQNTPIGD GPVLLPDNHY LSVQVVLSKD PNEKRDHMVL LEFRTAAGIT HGMDELYK
VFP2      IKANFKIRHN VEDGSVQLAD HYQQNTPIGD GPVLLPDNHY LSVQVVLSKD PNEKRDHMVL LEFRTAAGIT HGMDELYK
Sumire    IKANYKIRHN VEDGSVQLAD HYQQNTPIGD GPVLLPDNHY LSVQVVLSKD PNEKRDHMVL LEFRTAAGIT HGMDELYK
```

b

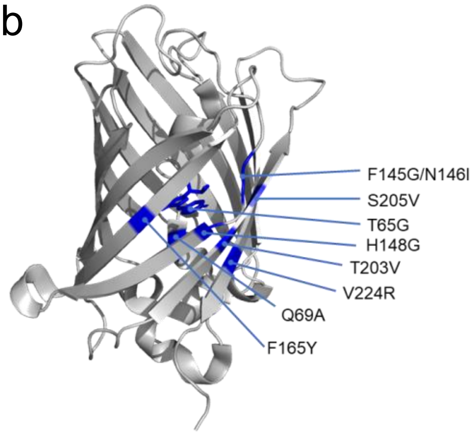

Supplementary Figure 1. The mutation points of Sumire. (a) Amid acid sequences of short wavelength mutants of avGFP. Mutations to avGFP are shown in black, and mutations introduced in this study are shown in blue. Chromophores are framed in red. (b) Mutational introduction site of Sumire on the crystal structure of sfGFP (PDB ID: 2b3p) (11)

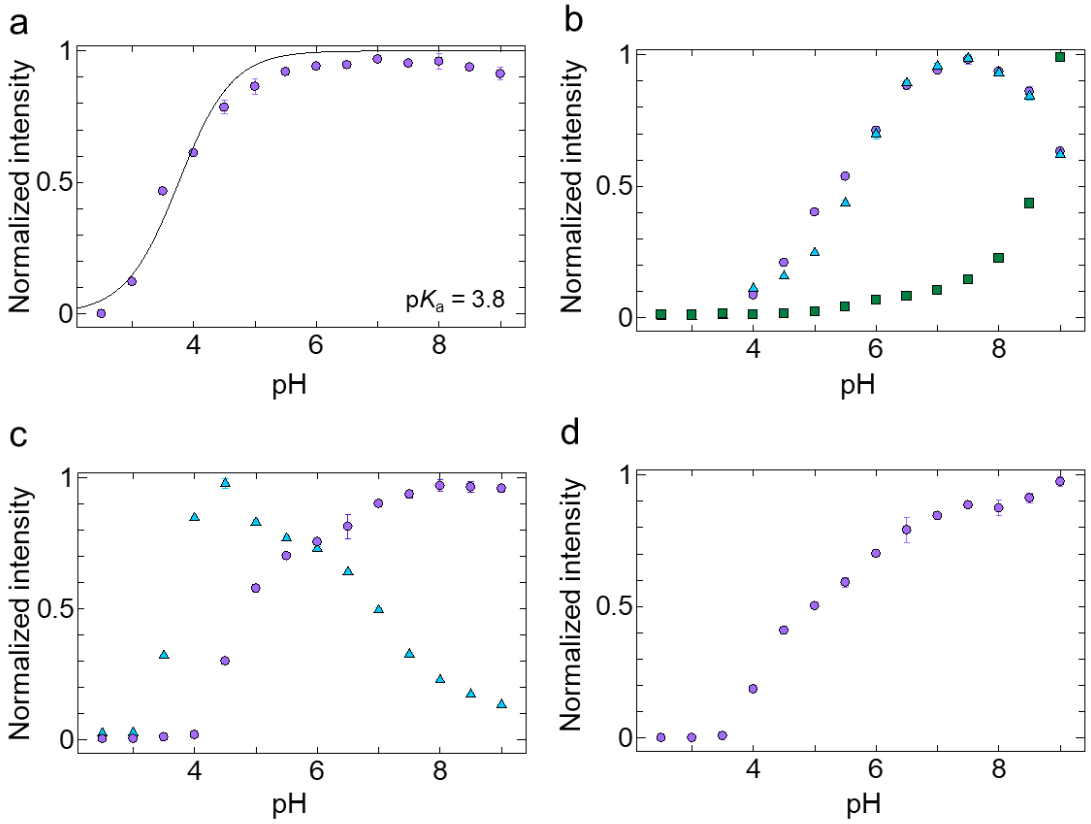

Supplementary Figure 2. The pH sensitivity of Sumire emission. The relative fluorescence intensities of Sumire (a), VFP0 (b), VFP1 (c) and VFP2 (d) were measured in 0.5 increments over a pH range of 3-9 in the buffer containing 30 mM trisodium citrate and sodium tetraborate, pH adjusted with HCl. Violet cycle, cyan triangle and green square indicate fluorescence intensities expected to be derived from hChr, nChr and iChr respectively.

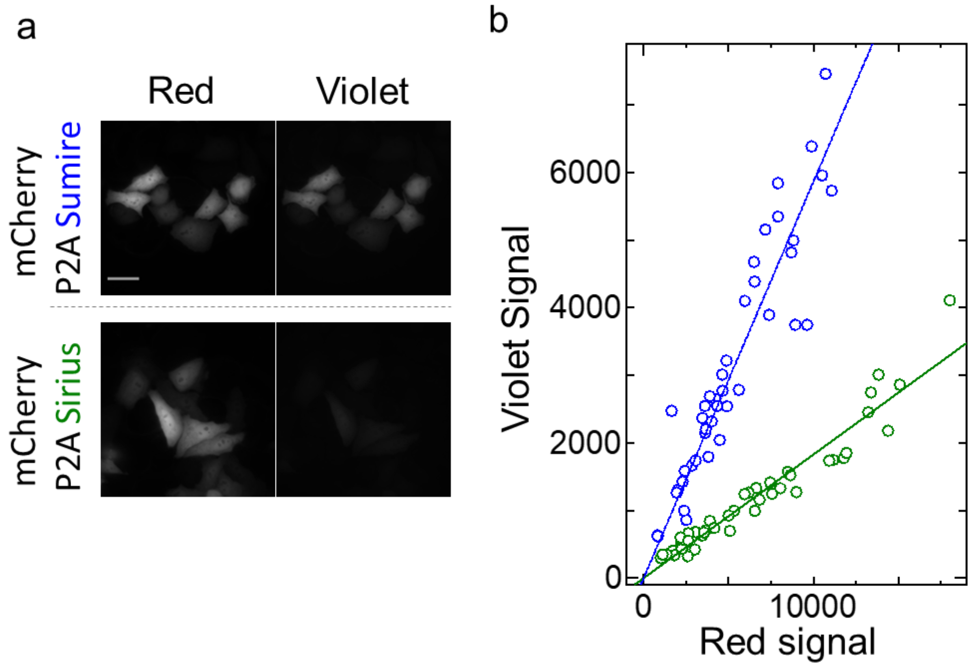

Supplementary Figure 3. Comparison of fluorescence intensity of Sumire and Sirius in HeLa cells using P2A assay. (a) Images of HeLa cells expressing mCherry P2A Sumire or Sirius under a microscope. Scale bar: 40 µm. (b) Signal intensity plots of violet (Sumire or Sirius) channel versus red (mCherry) channel. Solid line was an approximation using linear function of Sumire (blue) and Sirius (green). The slopes were 0.59 (Sumire) and 0.18 (Sirius). Three dishes each transfected with mCherry-P2A-Sumire and mCherry-P2A-Sirius were prepared and 42 and 45 cells were measured, respectively.

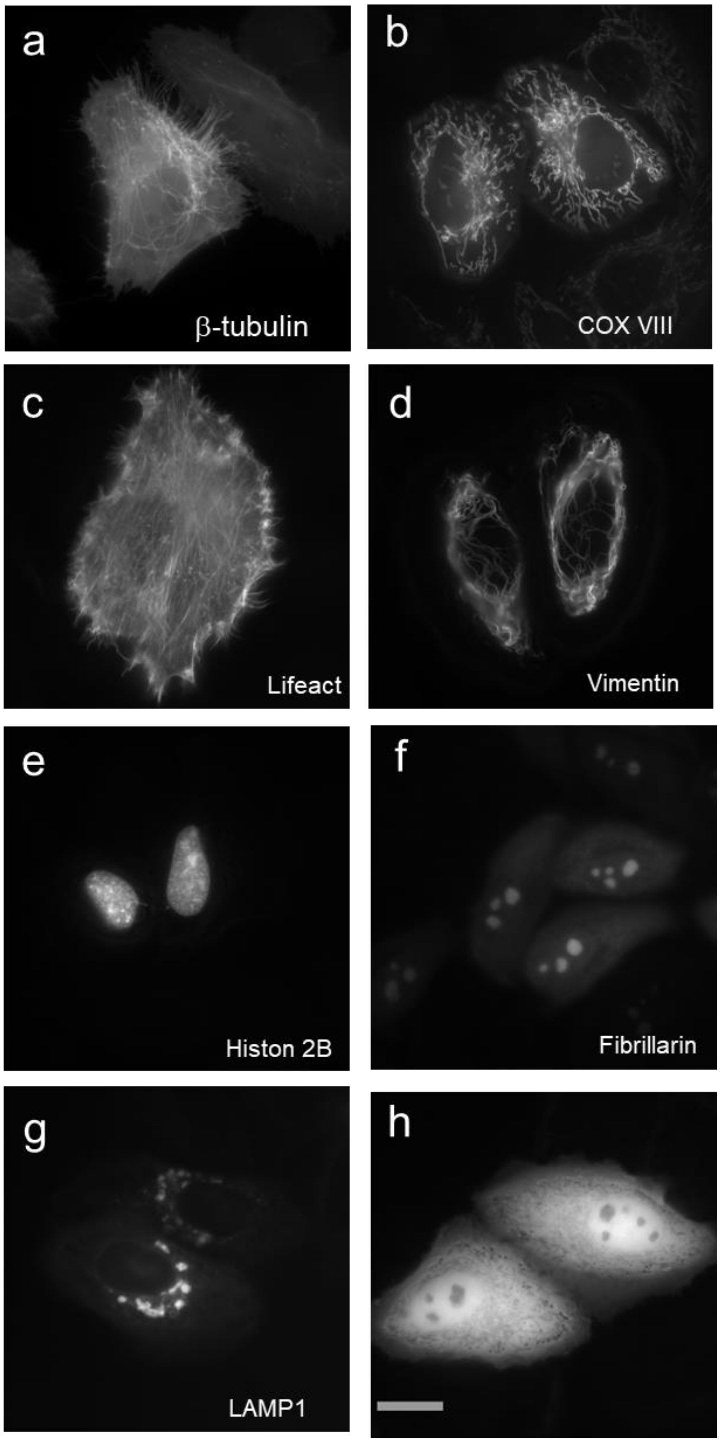

Supplementary Figure 4. Fluorescence localization. Fluorescence localization images of Sumire fused to (a) β-tubulin (mycrotubule) (b) CoxVIII signal peptide (mitochondria), (c) LifeAct (cytoskeleton), (d) vimentin (cytoskeleton), (e) H2B (nucleus), (f) fibrillarin (nucleoli), (g) LAMP1 (external part of lysosomes), and (h) non-fused sumire. Scale bars, 20 mm.

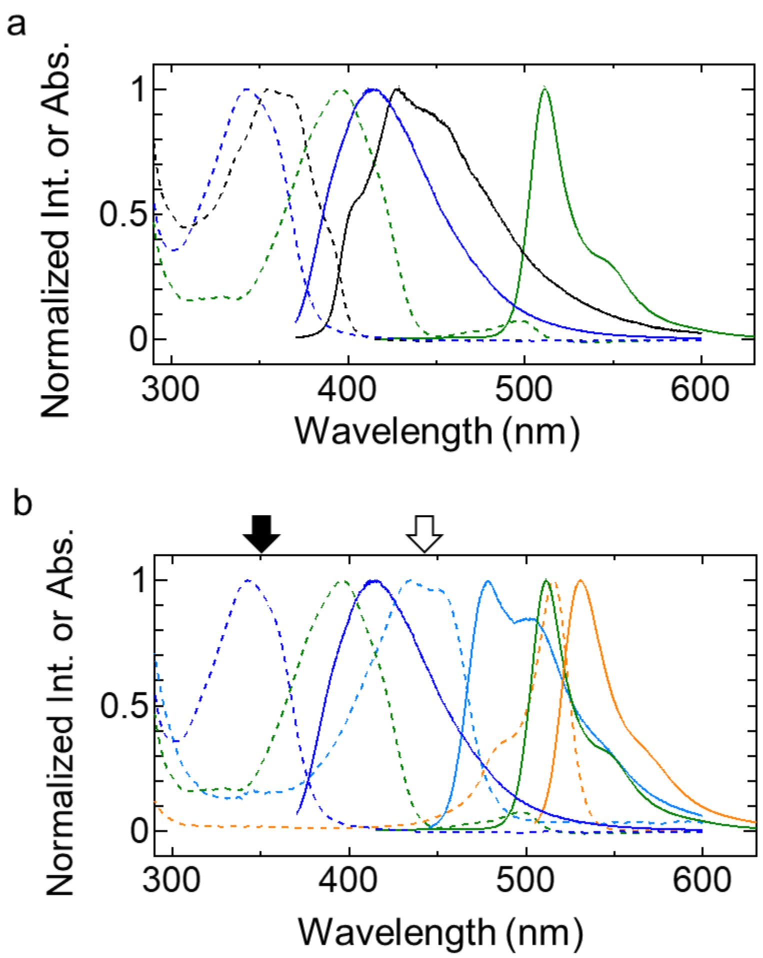

Supplementary Figure 5. Normalized spectra. (a, b) Normalized emission (solid line) and absorption spectra (dash line) of Sumire (blue), Sirius (black), T-Sapphire (green), ECFP (cyan), and Venus (yellow).

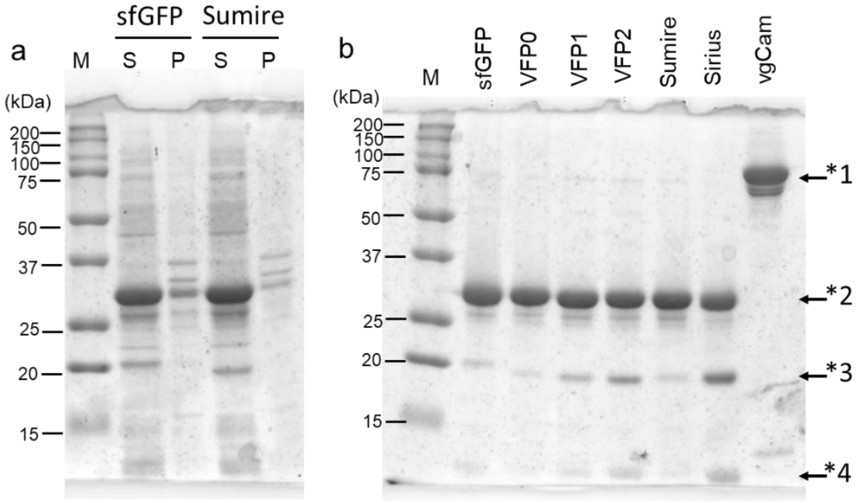

Supplementary Figure 6. Solubility and purity check. (a) Solubility check of Sumire. M: marker, S: supernatant, P: pellet. (b) 5 μg purified proteins were applied to the SDS page. *1: vgCam *2: FPs, *3 and *4: FP fragments expected to break the main chain in front of the chromophore.
