# Supplementary material for: Extension of the short wavelength side of fluorescent proteins using hydrated chromophores, and its application
Source: Commun Biol. 2022 Nov 3;5:1172. doi: 10.1038/s42003-022-04153-7 (PMC9633818; doi:10.1038/s42003-022-04153-7)
Supplement: Supplementary file 2 — Description of Additional Supplementary Files [file 42003_2022_4153_MOESM2_ESM.pdf]

Description of Additional Supplementary Files

File name: Supplementary Data 1
Description: Original data for Figs. 1a, 1b, 1c, 1d, 1e, 2b, 2d, S2a, S2b, S2c, S2d, S3b, S5a, and S5b.
